# Quantification of Total Phenolic and Carotenoid Content in Blackberries (*Rubus Fructicosus* L.) Using Near Infrared Spectroscopy (NIRS) and Multivariate Analysis

**DOI:** 10.3390/molecules23123191

**Published:** 2018-12-04

**Authors:** Eva María Toledo-Martín, María del Carmen García-García, Rafael Font, José Manuel Moreno-Rojas, María Salinas-Navarro, Pedro Gómez, Mercedes del Río-Celestino

**Affiliations:** 1 Department of Genomics and Biotecnology, IFAPA Center La Mojonera, Camino San Nicolás 1, La Mojonera, 04745 Almería, Spain; ortiztoledo@hotmail.com (E.M.T.-M.); pedro.gomez.j@juntadeandalucia.es (P.G.); 2 Department Agrifood Engineering and Technology, IFAPA Center La Mojonera, Camino San Nicolás 1, La Mojonera, 04745 Almería, Spain; mariac.garcia.g@juntadeandalucia.es; 3 Department of Food Science and Health, IFAPA Center La Mojonera, Camino San Nicolás 1, La Mojonera, 04745 Almería, Spain; rafaelm.font@juntadeandalucia.es; 4 Department of Food Science and Health, IFAPA Center Alameda del Obispo, 14080 Córdoba, Spain; josem.moreno.rojas@juntadeandalucia.es; 5 Department of Applied Biology (Genetic), University of Almería, Edificio CITE II-B, Ctra. Sacramento s/n, La Cañada de San Urbano, 04120 Almería, Spain; msalinas@ual.es

**Keywords:** blackberries, *Rubus fructicosus*, phenolics, carotenoids, bioanalytical applications, near infrared, chemometrics

## Abstract

A rapid method to quantify the total phenolic content (TPC) and total carotenoid content (TCC) in blackberries using near infrared spectroscopy (NIRS) was carried out aiming to provide reductions in analysis time and cost for the food industry. A total of 106 samples were analysed using the Folin-Ciocalteu method for TPC and a method based on Ultraviolet-Visible Spectrometer for TCC. The average contents found for TPC and TCC were 24.27 mg·g^−1^ dw and 8.30 µg·g^−1^ dw, respectively. Modified partial least squares (MPLS) regression was used for obtaining the calibration models of these compounds. The RPD (ratio of the standard deviation of the reference data to the standard error of prediction (SEP)) values from external validation for both TPC and TCC were between 1.5 < RPDp < 2.5 and RER values (ratio of the range in the reference data to SEP) were 5.92 for TPC and 8.63 for TCC. These values showed that both equations were suitable for screening purposes. MPLS loading plots showed a high contribution of sugars, chlorophyll, lipids and cellulose in the modelling of prediction equations.

## 1. Introduction

Consumers have high awareness of the health benefits of increased fruit and vegetable consumption, especially those rich in phytochemicals with nutraceutical properties. Vegetables and fresh fruit are reported to decrease the risk of cardiovascular diseases, certain forms of cancer and to prevent degenerative diseases [1,2]. This protection has been attributed to the fact that these foods may contain an optimal content of phytochemicals, such as antioxidants, fibre and other bioactive compounds [3]. These phytochemicals are in higher concentrations in small fruit, such as berries (blueberries, blackberries and strawberries) and this has motivated a large demand for fresh fruit. Berries are widely grown in Spain especially in Huelva (South-eastern Spain) where the cultivated area has increased in the last years to approximately 11,145 hectares in 2018, being more than 95% of national volume [4]. With reference to commercialization and sales prospects, the blackberry is the most promising reference [5,6].

Previous studies have reported that berry fruits contain a high total phenolic content (1.97–3.62 mg gallic acid equivalent GAE g^−1^ fresh weight (fw) and 16.94–31.13 mg GAE g^−1^ dry weight (dw) representing a rich source of antioxidants [5,7,8,9,10]. 

The total carotenoid content was also high in blackberry fruits with values from 0.86 µg·g^−1^ fw (7.4 µg·g^−1^ dw) to 21.40 µg·g^−1^ dw in blueberry fruits [11,12]. However, other authors also reported lower carotenoid contents ranging from 0.162 µg·g^−1^ fw (1.39 µg·g^−1^ dw) [13] to 1.84 µg·g^−1^ dw [14]. 

The demonstrated antioxidant capacity of blackberry fruits suggests that can play an important role against oxygen-free radical in the organism [15,16] and therefore for use in the development of functional food or nutraceuticals [17]. Due to the recognized importance of these antioxidant compounds, it is essential to characterize their content of them in the fruits.

Nowadays, the measuring of phenolic compounds and carotenoids is carried out using methods such as high performance liquid chromatography (HPLC) [18,19], gas chromatography (GC), or combinations of these methods with different systems of detection such as UV-Vis or mass spectrometry (MS) [20]. These methodologies are efficient for a rapid separation and quantification of these compounds. Although their use is common, these methods require sophisticated and expensive equipment, skilled labour and a variety of reagents which contain pollutants. Another relevant method includes spectrophotometry since it represents a relatively simple method for measuring phenolic compounds and carotenoids. As an alternative to these methods of analysis, NIRS (Near Infrared Reflectance Spectroscopy) technique offers several advantages such as high response, non-sample destruction, non-polluting and low analytical cost that does not require sophisticated sample preparation [21]. This methodology measures the interaction of the material with the light, which is in turn determined by the vibration of the chemical bonds of the sample constituents [22].

With regards to berry fruits, the studies with NIRS have been focused on determining the total phenolic content and antioxidant activity in intact berries (multispecies calibration) [23], in quality control and identification of food product adulteration of wild berry fruit extracts during storage [24], in evaluation of quality and nutraceutical compounds such as anthocyanin, polyphenol and flavonoid content of blueberries (*Vaccinium corymbosum* L.) [25] and also for detecting of an underground insect named *Eurhizococcus colombianus* (Hemiptera: *Margarodidae*) in blackberry leaves [26].

Since Andalusia (Southern Spain) is an important exporter of blackberries, there is interest in developing methodologies for the rapid analysis of antioxidant compounds as is demanded in both, food industries and in germplasm-screening programs. Nutritional quality improvement has been initiated in blackberry breeding programs; thus, rapid techniques such as those based on NIRS are needed for quick screening of lines with higher quality in early generations.

Therefore, the objectives of the present work were: (i) to study the potential of the NIRS technology for predicting the total phenolic and total carotenoid contents in blackberries, being these compounds constituting some of the main responsible molecules of the antioxidant properties in this fruit; (ii) to provide some knowledge about the mechanism used by NIRS for successfully determining these compounds in the fruits of this species.

## 2. Results and Discussion

### 2.1. Reference Analysis of Total Phenolic and Carotenoid Contents in the Samples

**Figure 1:**
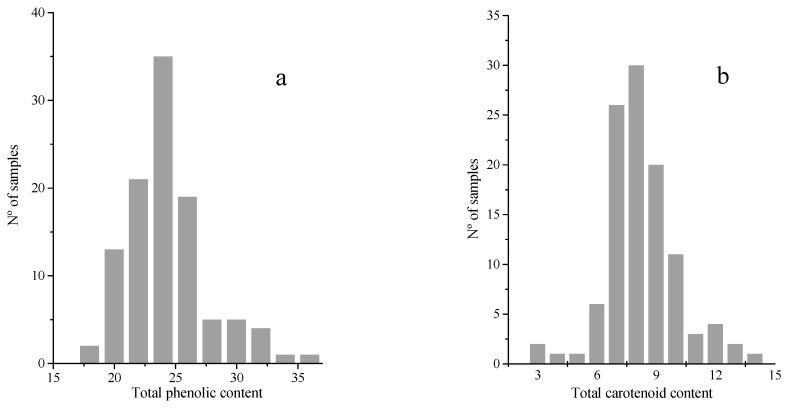
Frequency distribution plots for total phenolic (**a**) and total carotenoid content (**b**) by reference analysis.

Figure 1a,b showed frequency distribution plots of total phenolic and carotenoid contents for the samples (*n* = 106) used in this work, respectively. Such Total phenolic content (TPC) as Total carotenoid content (TCC) exhibited normal distributions in their intervals.

TPC values ranged from 17.36 to 35.67 mg·g^−1^ dw with mean and variation coefficient values of 24.27 and 13.67, respectively. These values were similar to those contents reported previously in studies carried out on blackberry fruits by Souza et al. [11] and Contessa et al. [10] with 34.53 and 36.78 mg·g^−1^ dw, respectively. Previous works have reported higher TPC concentrations (43.29–99.47 mg·g^−1^ dw) [23,27] and lower findings (5.58 mg·g^−1^ dw) [13,17] than those found in this study. The qualitative and quantitative differences found among fruits for the phenolic compounds could be due to factors such as environmental conditions, genotype, storage conditions and agro-techniques as observed by Aaby et al. [28] in berry fruits.

Regarding TCC, the values varied from 2.84 to 13.73 µg·g^−1^ dw with mean and variation coefficients of 8.30 and 21.92, respectively. Souza et al. [11] obtained similar results with 12.14 µg·g^−1^ dw of TCC in blackberries. Higher TCC contents (21.40 µg·g^−1^ dw) have been described in previous studies by Rutz et al. [13] and Lashmanova et al. [12].

### 2.2. Spectral Data Pre-Treatments and Equation Performances. Second Derivative Spectra of Blueberry Fruit

**Figure 2:**
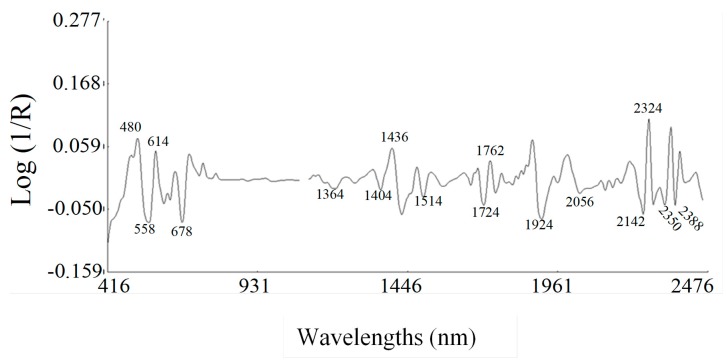
Second-derivative NIR spectra of blueberry samples.

Figure 2 shows the peaks and troughs corresponding to the points of maximum curvature in the raw spectrum. 

The bands in the visible region at 558 nm are due to electric transitions in the green, the band at 614 nm corresponding to electric transitions in the orange and 678 nm to electronic transitions in the red. The absorption band at 674 nm is assigned to absorption by chlorophyll [29]. 

The characteristic bands for phenolics can be observed in the NIR regions from 1415 nm to 1512 nm and from 1955 to 2035 nm [30]. The wavelength regions of the spectra in the ranges 1100–1250, 1300–1350 and 1650–1700 nm correspond to the 3rd overtone, the combination bands and the 1st overtone, respectively, of the C–H bonds of carotenoids [31]. In addition to these bands, the main absorption bands in the NIR segment of the spectrum were displayed at 1404 nm related to O-H stretch 1st overtone; at 1436 nm, which is characteristic of sugars [32] and related to combination O-H stretch/HOH deformation (O-H bend 2nd overtone; the band at 1724 nm related to lipid-specific 1st overtone [33]; at 1924 nm assigned to O-H stretch first overtone; the band at 2278 nm was assigned to the CH- stretch of cellulose [32]. The band at 2350 nm is related to C-H stretching first overtone of lipids, the peak at 2388 nm is associated with the C-H functional group present in hemicellulose and cellulose. Other absorptions were associated to the O-H 1st overtone (1364 nm) [33], the O-H group hydroxyl (1514 nm), C-O stretch of phenols (2056 nm), C-H stretch first overtone (1762 nm, 2142 nm and 2170 nm) [32].

### 2.3. Calibration Development for TPC and TCC

For calibration purposes, the wavelength ranges between 400–2500 nm were used. Table 1 summarizes the statistics of calibration and prediction models after the application of spectral pre-treatment. For the development of NIRS calibrations, four derivative mathematical treatments were tested: 1,4,4,1; 1,10,10,1; 2,5,5,2 and 2,20,20,2 (where the first digit is the number of the derivative, the second is the gap over which the derivative is calculated, the third is the number of data points in a running average or smoothing and the fourth is the second smoothing) [34]. The use of the second derivative to the raw spectra resulted in an increased complexity of spectra and assisted in a clear separation between peaks. 

The coefficient of determination for cross-validation (R^2^_CV_) for TPC was 0.70 in this study. The second derivative resulted in a better prediction in the cross-validation. This Modified partial least squares (MPLS) model (2,5,5,2) reached the best prediction precision. The number of latent variables was determined by cross validation of MPLS procedure and it was 8 for all models.

**Figure 3:**
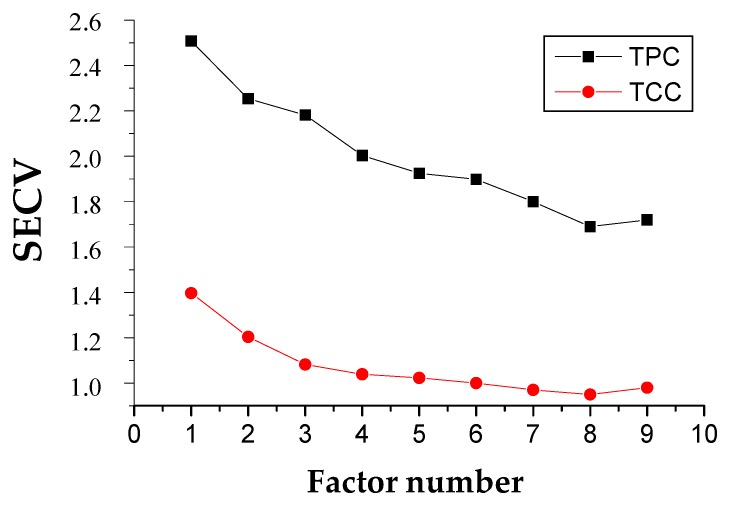
Plot of standard error of cross-validation (SECV) vs. the different number of factors included in the cross-validation of the modified partial least model for Total phenolic content (TPC) and total carotenoid content (TCC).

Figure 3 shows the plots of Standard error of cross-validation (SECV) versus the different number of factors included in the cross validation of MPLS for TPC and TCC models (2,5,5,2; standard normal variate and de-trending transformations (SNV + DT)). The number of factor of 8 were optimum for both parameters as it resulted in the minimum SECV of 1.69 and 0.95 for TPC and TCC models, respectively. This SECV value was close to the value of SEC which then shows that the calibration carried out was feasible.

Other authors have shown the ability of NIRS to predict the content of phenolic compounds in blueberries (*Vaccinium corymbosum* L.) [25], in methanolic extracts of berry fruits (wild blueberries, blackberries, raspberries, strawberries and red currants) reporting high R^2^ coefficients (ranging from 0.864 to 0.975). 

R^2^_CV_ and RPD_CV_ coefficients for the cross-validation (treatment 2,5,5,2) were 0.76 and 1.91, respectively for total carotenoid content in blackberry fruits. 

### 2.4. External Validation

Table 2 shows the external validation statistics (SEP, Q^2^, RPDp and RER) for both compounds measured in blackberries. 

The SEP values in the external validation were lower than their respective standard deviation, which point that NIRS is able to determine these traits in blackberry fruits. 

The Q^2^ values give an indication of the percentage variation in the Y variable that is accounted for by the X variable. Therefore, Q^2^ values above 0.50 indicate that over 50% of the variation in Y is attributable to variation in X and this allows discrimination. In our study, external validation resulted in Q^2^ of 0.65 and 0.71 for TPC and TCC respectively (Table 2) which indicated that 65% and 71% of the variability in the data was explained by the respective calibration model.

According to Williams and Norris [35], the values for Q^2^ obtained from the external validations in this work, indicated that the models for both TPC and TCC can be classified as models that can be used for rough predictions of samples (Table 2).

The Q^2^ statistic obtained in external validation for TPC was lower than those reported in previous works. Sinelli et al. [25] indicated a Q^2^ value of 0.87 for TPC in blueberry fruits. Other authors found Q^2^ values of 0.98 for different berry species [23]. According to the guidelines for interpretation of RPDp from external validation [35], if this ratio is between 1.5 < RPDp < 2.5 this characterizes the equations as suitable for screening purposes, which was obtained for TPC (1.52) and TCC (1.82). Similar results were obtained in blueberry [25] with RPDp = 2.05. However, a higher RPDp value (RPDp = 3.05) has been reported for different berry species than those found in this work [23]. 

**Figure 4:**
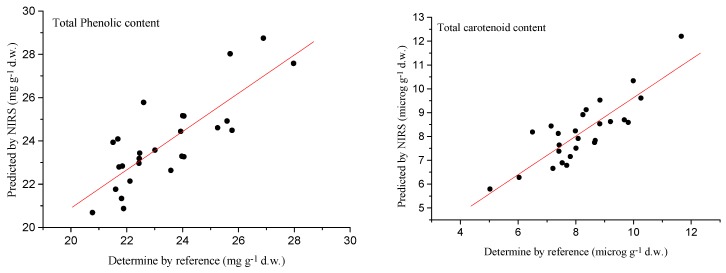
External validation scatter plot for near infrared predicted values versus reference values for total phenolic content (TPC) and total carotenoid content (TCC) in blackberries.

The results were corroborated by the Figure 4 of predicted values versus reference values obtained using the MPLS model (second derivative) for the TPC and TCC validation sets. 

The calibration equation obtained in the present work for TPC showed an RPDp value lower than that reported by Gajoš [23], which developed a multicalibration equation for wild blueberries, blackberries, raspberries, strawberries and red currants. This could be due to the narrower range of TPC content found in the samples of our study which varied from 20.77 to 27.97 mg·g^−1^ dw (Table 2). As the RPDp value is highly dependent on the range of the sample population for a determined parameter [36], a wider range implies a higher RPD value. 

In terms of RER coefficients, predictive ability of the prediction models in this work ranged from 5.92 to 8.63. For TPC and TCC, the validation yielded RER (5.92 and 8.63, respectively) values which indicated models that can be used for screening purposes, thus being very useful in quality control and as a selection tool in blackberry breeding programmes [36]. 

### 2.5. Modified Partial Least Square Loadings for Total Phenolic Content

MPLS regression was used to obtain the spectral information and predict the sample composition. 

**Figure 5:**
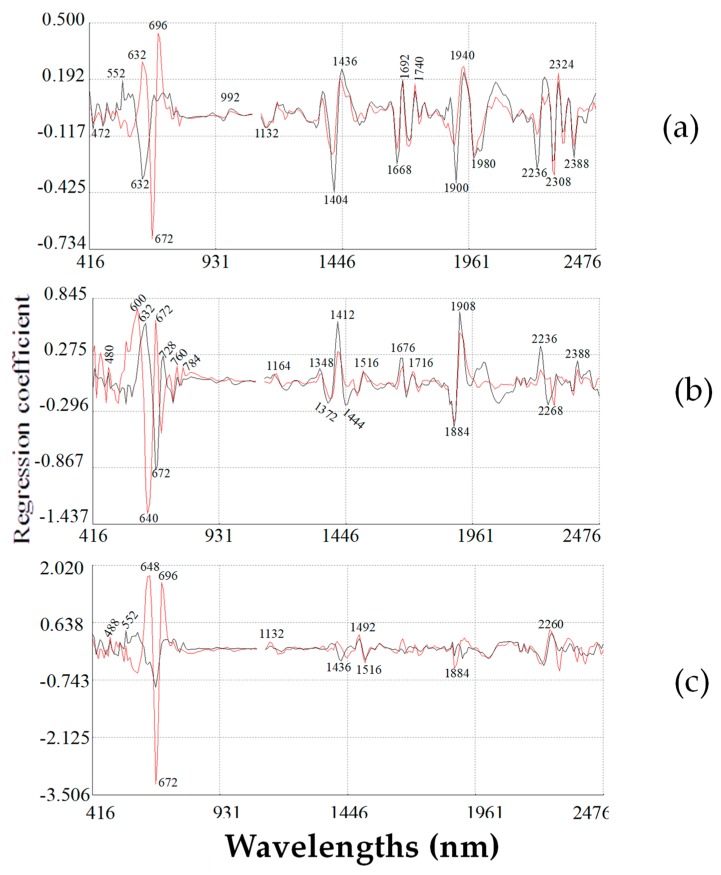
MPLS loading plots for TPC (black line) and TCC (red line) using near-infrared reflectance spectroscopy. (**a**) First loading; (**b**) Second loading; (**c**) Third loading.

Figure 5 shows the equation corresponding to TPC. Some of the spectral regions used by the TPC models for calibrating these compounds have been previously reported by other authors [32]. The first MPLS term was influenced by absorption bands characteristic of electronic vibrations at 632 nm, it was also influenced by absorption bands at 1412 and 1668 nm. Vibration differences in the range 1399–1699 nm have been identified for fruit products such as wine, grape juice and orange fruit [21,37,38] presenting the vibration range of the C-H and O-H bonds, corresponding to water and phenolic absorbance [21]. There was also a peak at 1908 nm influenced by absorption assigned to the first OH stretch. At 1980 nm it corresponded to C-H aromatic 2nd overtone, it also relate to one or more aromatic rings and hydroxyl groups, mainly related to combination bands of the -OH functional group, symmetric and anti-symmetric stretching. The absorption vibrations at 2236, 2300 and 2388 nm were due to N-H bend [32]. The second and third terms were influenced by absorption bands characteristic to electronic vibrations at 640 nm and 672 nm, respectively. 

### 2.6. Modified Partial Least Square Loadings for Total Carotenoid Content

MPLS loading plots of the TCC equation are shown in Figure 5. The first term (Figure 5a) was influenced by bands which corresponding to electronic vibration assigned to chlorophyll at 672 nm [39], second C=O stretch at 1900 nm. Absorptions at 1980 nm and 2300 nm correspond to vibrations in N-H stretch bending and C-H combination tones by lipids [32]. 

Those wavelengths corresponding to absorptions by electronic vibration assigned to chlorophyll (672 nm) and stretch groups: C=O and O-H (1444 nm), C-O (1692 nm), N-H with C-O (2068 nm) and OH cellulose stretch (2268 nm) highly influenced the second factor of the equation (Figure 5b). The third term (Figure 5c) of the equation was modelled with those wavelengths corresponding to electronic vibrations (672 nm) with the following stretches: C=O (1420 nm), N-H (1516 nm) and O-H (1908 nm) [32].

## 3. Materials and Methods

### 3.1. Plant Material and Greenhouse Experiments

Blackberry (*Rubus fructicosus* L.) cv. Tupy was chosen for the field trials. 

The plant transplant took place on November 29th 2013 in a greenhouse of 600 m^2^ in the IFAPA Centre La Mojonera, Almería (36°47′19″ N, 02°42′11″ W; 142 m a.s.l.), following standard cultural practices for disease control, insect pest and plant nutrition. 

The blackberry plants were transplanted on polypropylene containers of 15 l capacity using coconut fibre substrate. The irrigation water conditions were pH 8.1 and 1.26 mS·cm^−1^ conductivity and the nutrient solution had pH 5.8 and 2.50 mS·cm^−1^ electrical conductivity.

**Figure 6:**
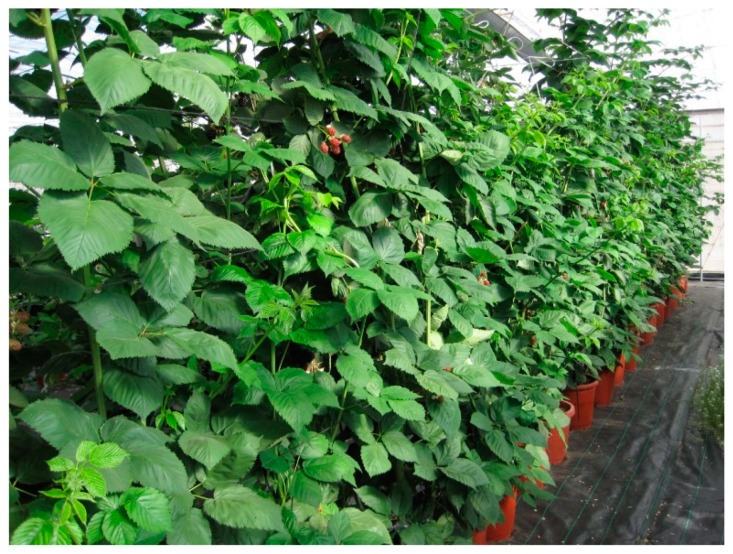
Trial of Tupy blackberry variety growing in greenhouse.

The trial (Figure 6) was designed as a randomized complete block with 3 replicates and 20 plants per repetition. Thirty fruits were collected per each plant and stored at −80 °C until lyophilization, then were lyophilized (Telstar LyoQuest, Terrassa, Spain) and ground in a mill (Janke & Kunkel, model A10, IKA^®^-Labortechnik). The samples were lyophilized to remove the strong absorbance of water in the infrared region, which overlaps with important bands of nutritional parameters present in low concentration [40].

Two samplings were performed at the time of maximum production (21 April 2014 and 20 May 2014). 

The fruits harvested were classified according to their colour with a colorimeter to avoid fruit-to-fruit variation in ripeness, thus these were considered to be ripe when the CIE L*a*b (CIELAB) values were L: 21.11; a: 0.835; b: 0.073 y C: 1.27.

### 3.2. Determination of the Total Phenolic Fraction

Five grams of each sample (fresh weight) were homogenized in 20 mL of ethanol (99.7%) and stored at −20 °C for 2 weeks. An aliquot of 60 µL supernatant was taken previous to centrifugation of the extracts and then prepared according to the modified method by Dewanto et al. [41]. After 75 min, the absorbance was measured at 765 nm using a Thermo Spectronic UV–visible Spectrometer (Thermo Fisher Scientific, Waltham, MA, USA). The external standard gallic acid, 3,4,5-trihydroxybenzoic acid (Sigma–Aldrich, Steinheim, Germany) was used for quantifying. The results were expressed in mg GAE (gallic acid equivalent) g^−1^ dry weight.

### 3.3. Determination of the Total Carotenoid Content

Analysis of total carotenoid content was carried out by the method described by Rutz et al. [13].

Five grams of each sample and 2 g of celite were added to 20 mL of cold acetone and the mixture was shaken for 10 min. The material was filtered with a Buchner funnel with filter paper, washing the sample with acetone until the extract was colourless. The filtrate was transferred to a separatory funnel, to which 30 mL of petroleum ether and 30 mL of distilled water were added. The lower phase was discarded, then distilled water was added; this procedure was repeated four times to achieve total removal of the acetone. The upper phase was transferred to a 50-mL volumetric flask and the volume was completed with petroleum ether. The absorbance was measured with a Thermo Spectronic UV–visible Spectrometer (Thermo Fisher Scientific, Delaware, USA) at 450 nm, using petroleum ether as a blank. The total carotenoid content was determined by Equation (1) and the results were expressed in mg of total carotenoids per g dry weight. 

### 3.4. NIRS Analysis Calibration and Validation Development

One hundred and twenty freeze-dried blackberry samples were analysed by NIRS (90 calibration, 30 calibration). An spectrometer (Model 6500 Foss-NIRSystems, Inc., Silver Spring, MD, USA) was used for registrating the spectra in the range from 400–2500 nm each 2 nm in reflectance mode.

Freeze-dried, ground samples of the blackberries were placed in the sample holder (3 cm diameter, 10 mL volume approximately) until it was full (sample weight: 3.50 g) and then were scanned. Their spectra were acquired at 2 nm wavelength resolution as log 1/R (R is reflectance) over a wavelength range from 400 to 2500 nm (visible and near-infrared regions).

The spectral variability and structure of the sample population was performed using the CENTER algorithm; samples with a statistical value >3 were considered anomalous spectra or outliers [42]. 

Calibration equations for total phenolic content and total carotenoid content were developed on the whole set (*n* = 90) using the application GLOBAL v. 1.50 (WINISI II, Infrasoft International, LLC, Port Matilda, PA, USA). Calibration equations were computed using different mathematical treatments although only those that displayed the higher predictive capacity were showed: [(1,4,4,1); (1,10,10,1); (2,5,5,2); (2,20,20,2)] where the meaning of each term is the derivative order of the log 1/R data (being R the reflectance), segment of the derivative, first smooth and second smooth). Additionally to the use of derivatives, standard normal variate and de-trending (SNV-DT) transformations [43] were used, which are algorithms used to correct baseline offset due to scattering effects (differences in particle size and path length among samples) and improve the accuracy of the calibration.

Modified partial least squares (PLSm) was used as a regression method to correlate the spectral information (raw optical data or derived spectra) of the samples and TPC and TCC contents determined by the reference method, using different number of wavelengths from 400 to 2500 nm for the calculation. The objective was to perform a linear regression in a new coordinate system with a lower dimensionality than the original space of the independent variables. The PLS loading factors (latent variables) were determined by the maximum variance of the independent (spectral data) variables and by a maximum correlation with the dependent (chemical) variables. The model obtained used only the most important factors, the “noise” being encapsulated in the less important factors. 

Cross-validation was performed on the calibration set to determine both, the ability to predict on unknown samples and the best number of terms to use in the equation [44]. The number of principal component terms used in the equation to explain the analyte variance was also taken into account before selecting the equation for use. The cross validation process used in the software should prevent over fitting of the equation to the calibration set as the optimum number of terms are selected when the SECV is at its lowest and R^2^_CV_ is at its highest. Addition of more terms than necessary will increase the prediction error and over fit the equation to its calibration set resulting in poor predictive performance on samples outside the calibration set. Usually a medium sized model is preferred. An external validation in 30 independent samples was carried out to evaluate the accuracy and precision of the calibration equations for total phenolic and carotenoid content following the protocol outline by Shenk et al. [44]. The 30 samples of the validation set were selected by taking one of every 5 samples in the 120 samples set; finally, the calibration set was constituted of the 90 remaining samples. The standard error (SE) and coefficient of determination were calculated for cross-validation (R^2^_CV_) and external validation (Q_2_). The predictive ability of the equations was assessed in the external validation from the Q^2^ coefficient, the RPD (the ratio of the standard deviation for the samples of the validation to the SEP (standard error of prediction (performance) and the RER (the ratio of the range in the reference data (validation set) to the SEP). NIR models can be classified depending the Q^2^ from the external validation [36] as: if 0.26 < Q^2^ < 0.49, the models show a low correlation;); if 0.50 < Q^2^ < 0.64) models can be used for rough predictions of samples; if 0.65 < Q^2^ < 0.81) the models can be used to discriminate between low and high values of the samples; (if 0.82 < Q^2^ < 0.90 are models with good prediction; if Q^2^ > 0.90 the models show excellent precision. RPD values > 3 are desirable for excellent calibration equations, however equations with an RPD < 1.5 are unusable [35]. The RER (ratio of the range to standard error of prediction (performance), it should be at least 10 [36].

The mathematical expressions of these statistics are as follows:

$$\mathrm{RPD}=SD\langle {\left[\left(\sum_{i=1}^{n}{\left(y_{i}-{\hat{y}}_{i}\right)}^{2}\right){\left(N-K-1\right)}^{-1}\right]}^{1/2}\rangle^{-1}$$

where $y_{i}$ = laboratory reference value for the *i*th sample; $\hat{y}$ = NIR value; *K* = number of wavelengths used in an equation; *N* = number of samples; *SD* = standard deviation.

$$\mathrm{RER}=range\langle {\left[\left(\sum_{i=1}^{n}{\left(y_{i}-{\hat{y}}_{i}\right)}^{2}\right){\left(N-K-1\right)}^{-1}\right]}^{1/2}\rangle^{-1}$$

where $y_{i}$ = laboratory reference value for the *i*th sample; $\hat{y}$ = NIR value; *K* = number of wavelengths used in an equation; *N* = number of samples.

## 4. Conclusions

The NIRS technique has the potential to reduce the cost and time in analysing the total phenolic and carotenoid content in blackberries for both agri-food applications and research. Approximately each 1.5 min we can analyse a sample for both quality components by using the NIR spectroscopy. From the different mathematical treatments tested the second derivative produced the better results for predicting, however the models reported here are usable for routine screening of a large number of samples in breeding programs. 

The spectral regions corresponding to absorbance by cellulose, lipids, chlorophyll and sugars were used by MPLS for modelling the prediction equations for total phenolic and carotenoid content in blackberries.

## Figures and Tables

**Table 1:** Calibration and cross-validation statistics of total phenolic content (TPC expressed as mg·g^−1^ dw) and total carotenoid content (TCC expressed as µg·g^−1^ dw) for blackberry fruit measured by FNS-6500 with different treatments.

Trait	Range	SD ^a^	R^2 b^	SEC ^c^	R^2^_CV_^d^	SECV ^e^	RPDcv ^f^	Treatment	Factor ^g^
TPC	17.36–35.67	3.06	0.86	1.14	0.69	1.69	1.81	2,5,5,2	8
	17.36–35.67	3.06	0.71	1.66	0.59	1.95	1.58	1,4,4,1	8
	17.36–35.67	3.06	0.70	1.68	0.59	1.97	1.57	1,10,10,1	8
	17.36–35.67	3.06	0.76	1.49	0.67	1.75	1.75	2,20,20,2	8
TCC	2.84–13.73	1.82	0.92	0.52	0.76	0.95	1.91	2,5,5,2	8
	2.84–13.73	1.82	0.85	0.72	0.71	1.03	1.83	1,4,4,1	8
	2.84–13.73	1.82	0.83	0.75	0.71	0.99	1.82	1,10,10,1	8
	2.84–13.73	1.82	0.84	0.75	0.70	1.05	1.80	2,20,20,2	8

^a^ SD: standard deviation; ^b^ R^2^: coefficient of determination in calibration, ^c^ SEC: standard error in calibration, ^d^ R^2^_CV_: coefficient of determination in cross-validation, ^e^ SECV: standard error of cross-validation, ^f^ RPDcv: ratio of the standard deviation to standard error of cross-validation; ^g^ Factor: number of latent variables.

**Table 2:** Reference values and external validation statistics of the NIRS calibrations for total phenolic content (TPC expressed as mg·g^−1^ dw) and total carotenoid content (TCC expressed as µg·g^−1^ dw) in blackberry fruit.

	Reference Values (*n* = 30)				External Validation		
Parameters	Range	Mean	SD ^a^	Q^2 b^	SEP ^c^	RDPp ^d^	RER ^e^
TPC	20.77–27.97	23.41	1.85	0.65	1.22	1.52	5.92
TCC	5.02–11.66	8.21	1.40	0.71	0.77	1.82	8.63

^a^ SD: standard deviation; ^b^ Q^2^: coefficient of determination in external validation; ^c^ SEP: standard error of prediction corrected for bias; ^d^ RPDp: ratio of the standard deviation to standard error of prediction (performance); ^e^ RER: ratio of the range to standard error of prediction (performance).
